# Tailoring of structural, morphological, electrical, and magnetic properties of LaMn_1−*x*_Fe_*x*_O_3_ ceramics

**DOI:** 10.1039/d4ra04931d

**Published:** 2024-07-29

**Authors:** Priyanka Thakur, Kais Iben Nassar, Deepak Kumar, Pawan Kumar, Prianka Sharma, Vineet Tirth, Ali Saad Alosaimy, Ali Algahtani, Manel Essid, Madan Lal

**Affiliations:** a Department of Physics, Akal College of Basic Sciences, Eternal University Baru Sahib HP-173101 India madan.physics26@gmail.com; b Department of Physics, 3N-Aveiro, University of Aveiro 3810-193 Aveiro Portugal; c Department of Physics, Graphic Era (Deemed to be University) Clement Town Dehradun UK-248002 India; d Department of Physics, School of Basic & Applied Sciences, Maharaja Agrasen University HP 174103 India; e Department of Mechanical Engineering, College of Engineering, King Khalid University Abha 61421 Asir Kingdom of Saudi Arabia; f Research Center for Advanced Materials Science (RCAMS), King Khalid University Guraiger Abha-61413 Asir Kingdom of Saudi Arabia; g Department of Mechanical Engineering, College of Engineering, Taif University Taif 21944 Kingdom of Saudi Arabia; h Department of Chemistry, College of Science, King Khaled University (KKU) P.O. Box 9004 Abha 61413 Saudi Arabia

## Abstract

This study undertakes a comparative analysis of the structural, morphological, electrical, and magnetic characteristics of Fe-doped LaMnO_3_ ceramics. The solid-state reaction method was used to prepare Fe-doped LaMnO_3_ at different concentrations (0.00 ≤ *x* ≤ 1.00) and has been characterized using X-ray diffraction (XRD), Fourier transforms infrared spectroscopy (FTIR), Field emission scanning electron microscopy (FE-SEM), energy-dispersive spectroscopy (EDS), and vibrating sample magnetometry (VSM). The structural transformation from rhombohedral to orthorhombic with Fe-doping is demonstrated by Rietveld's refined XRD patterns. The positive slope in Williamsons–Hall's (W–H) plots confirms the presence of tensile strain with increasing average crystallite size. Quasi-spherical morphology of all the compositions with similar uniformity was confirmed by FESEM images. The chemical distribution of all the elements has been identified by EDS mapping images. Normal dielectric dispersion behaviour of all the samples with NTCR response is confirmed by dielectric and impedance analysis respectively. Increasing lattice volume with Fe-concentration results is increasing *E*_a_. The presence of antiferromagnetic ordering, in addition to weak ferromagnetic ordering, is indicated by the unsaturated magnetization even up to a high external field. The decrease in *M*_S_ and increase in *H*_C_ values due to Fe-doping reflect the influence of particle size on various magnetic parameters.

## Abstract 2

This study undertakes a comparative analysis of the structural, morphological, electrical, and magnetic characteristics of Fe-doped LaMnO_3_ ceramics.

## Graphical abstract

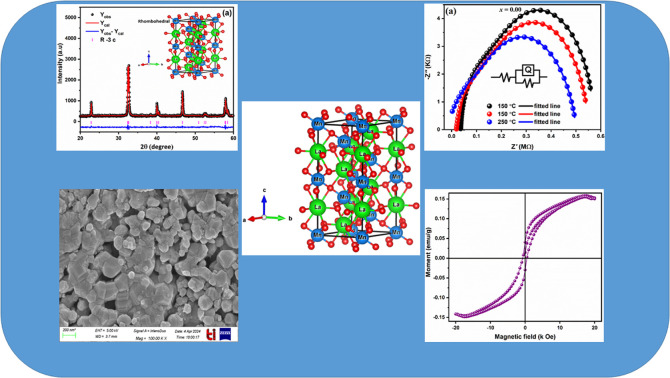

## Introduction

Perovskite materials based on lanthanum have been widely used in numerous applications because of their apparent structural, electrical, optical, catalytic, and magnetic capabilities.^1–4^ Their structures' substitution of metals, varying oxidation states, variable oxygen stoichiometry, and stability of these materials show their wide applicability in solid oxide fuel cell electrodes,^5^ chemical sensors,^6^ optoelectronic devices,^7^ spintronics, and solar cells,^8^ because of their low power consumptions. The features of lanthanum-based perovskite materials' microwave absorption have been extensively researched to solve issues connected to radiation pollution and electromagnetic interference (EMI), which pose a threat to information security as well as human health and device operation.^9–11^

Lanthanum-based perovskite materials such as LaMnO_3_, LaFeO_3_, LaCrO_3_, LaNiO_3_, and LaCoO_3_, have been widely studied due to their ease of manufacture, flexibility, and low production cost. LaMnO_3_, and LaFeO_3_, are perovskite-type materials generally having orthorhombic crystal structures at room temperature. LaMnO_3_ (LMO) is an A-type antiferromagnetic perovskite material that shows orthorhombic crystal symmetry only around its stoichiometric composition.^12–14^ The magnetoresistance properties of LMO make it an important multiferroic material that can be exploited for the use of LMO as electrode material in supercapacitors.^15^ On the other hand, LaFeO_3_ (LFO) is an orthoferrite, has a Néel temperature (*T*_N_) of roughly 738 K, and a G-type antiferromagnetic structure.^9,16^ Orthorhombically distorted LFO-perovskite was found to have multiferroic properties and has potential applications as a spin filter in spintronics.^17,18^

It has been observed that 3d cations on the B-site (*i.e.*, Mn^4+^ or Fe^3+^) of the crystal lattice drive the electrical and magnetic properties of LMO and LFO. The double exchange mechanism (DE) and the Jahn–Teller effect are the two main reasons for the co-existence of two ferroic orders in LMO and LFO ceramics. DE mechanism is the hopping of e_g_ electrons between Mn^3+^ and Mn^4+^ mediated by oxygen anions, while the Jahn–Teller effect is strong electron-phonon coupling arises due to the deformation of [MnO_6_] or [FeO_6_] octahedral. These two machines were observed in lanthanum-based ceramics by appropriate doping or by introducing oxygen vacancies in the crystal structure.^19–21^ Lanthanum is a non-magnetic rare earth metal with zero unpaired electrons in its La^3+^ oxidation state which does not influence magnetic properties directly.^22^ Hence, due to the presence of unpaired electrons and high magnetic moment values of B-site ions (Fe and Mn ions), a change in magnetic properties has been observed in lanthanum-based perovskite materials. Thus, it is important to study the Mn-site substitution in LaMnO_3_ as the Mn-site doped ion will consequently result in the distortion of the Mn–O plane, which will cause a considerable change in structural and electrical and magnetic properties.^23^ In recent years, extensive research has been conducted on the effects of substituting Mn sites with various cations (M = Fe, Cr, Co, Ni) in both charge-delocalized and localized manganite's.^24–27^ Among these, Fe ions, due to their close ionic radii to Mn^3+^, are particularly interesting as dopants for LaMnO_3_, as they can occupy Mn sites as Fe^3+^ without causing significant lattice distortion.^28^

Different groups have reported on a number of investigations including Fe doping at the Mn location.^29–32^ With decreasing temperature, the perovskite system La_0.67_Ca_0.33_Mn_1−*x*_Fe_*x*_O_3_ (*x* = 0.00, 0.01, 0.03, and 0.07) exhibits an essentially paramagnetic (PM) to ferromagnetic (FM) transition, and as Fe concentration increases, the PM to FM transition temperature is decreased.^32^ Magnetization measurements of the system La_0.6_Ca_0.4_Mn_1−*x*_Fe_*x*_O_3_ (*x* = 0, 0.05, 0.1, 0.15, and 0.2) revealed that the coexistence of ferromagnetic and antiferromagnetic interactions for critical composition *x* ∼0.1 and that the Curie temperature (*T*_C_) decreases upon Fe-doping from 275 K to 75 K for *x* = 0 and *x* = 0.2, respectively.^31^ In their studied system La_0.7_Ca_0.3_Mn_1−*x*_Fe_*x*_O_3_ (*x* = 0.08, 0.1, and 0.12), Sahasrabudhe *et al.*^33^ found only one ferromagnetic phase exist below the transition temperature without any spin glass behaviour. Kundaliya *et al.*^34^ also noted comparable outcomes for the combination La_0.67_Ca_0.33_Mn_0.9_Fe_0.1_O_3_. Fe-doping of La_1−*x*_Ca_*x*_MnO_3_ results in the suppression of ferromagnetism and conduction in both the ferromagnetic (*x* = 0.37) and antiferromagnetic (*x* = 0.53) phases.^35^ This is because double-exchange interactions are reduced and the number of hopping electrons is reduced. In the La_0.67_Ca_0.33_Mn_0.9_Fe_0.1_O_3_ perovskite, the random substitution of Fe^3+^ with Mn^3+^ lowers the number of locations where the Mn e_g_ (up) electron can hop, which lowers ferromagnetic exchange. At low temperatures, the system is driven into a randomly canted ferromagnetic state by the competition between the co-existing antiferromagnetic super-exchange interactions and the ferromagnetic double-exchange interactions.^36^

Fe^3+^ can readily take the place of Mn^3+^ in the MnO_6_ octahedron. Reduced Jahn–Teller distortion from Mn^3+^ ions results from partial replacement of Fe^3+^ for Mn^3+^. In the meantime, resistivity rises as a result of Fe doping, which lessens the double exchange contact between Mn^3+^ and Mn^4+^. The degree of the double exchange effect's destruction deepens, the degree of the crystal structure's distortion is lessened, the symmetry of the crystal structure is strengthened, the magnetoresistance (MR) progressively rises, and the temperature coefficient of resistance (TCR) initially rises and then falls with an increase in Fe doping.^37–39^ Kholil *et al.*,^40^ reported in their report that Fe-doping can be used to reduce bandgap in perovskites and also shift the optical conductivity in the visible region. Thus, Fe-doped perovskites provide promising candidates for applications in optoelectronics, solar cells, solid oxide fuel cells and in fabrication of new magnetic devices.^41–43^

Numerous studies have investigated Fe-doped LaMnO_3_; however, none have explored the complete substitution of Mn ions with Fe-ions. Thus, this study offers an extensive examination of the structural, morphological, magnetic, and electrical characteristics of LaMn_1−*x*_Fe_*x*_O_3_ ceramics. In addition to examining how magnetic behaviour changes with different Fe doping concentrations in LaMnO_3_, we also analysed dielectric and impedance characteristics at various temperatures and frequencies.

## Results and discussion

**Fig. 1:**
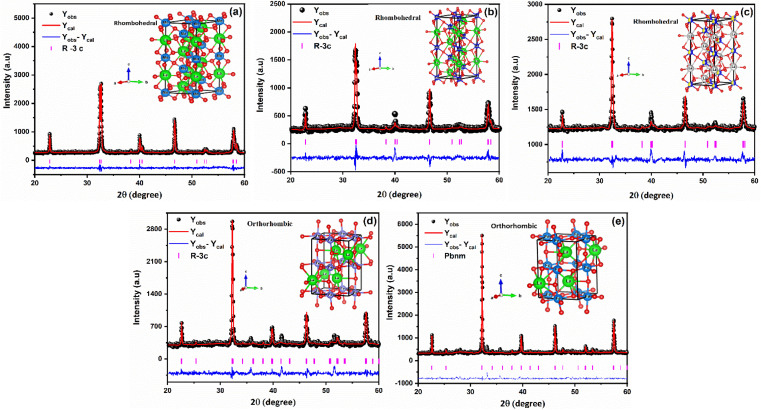
Rietveld refined XRD pattern of LaMn_1−*x*_Fe_*x*_O_3_ ceramics (where (a) *x* = 0.00, (b) *x* = 0.25, (c) *x* = 0.50, (d) *x* = 0.75, (e) *x* = 1.00).

FullProf software was utilized to process the Rietveld refinement of XRD data for LaMn_1−*x*_Fe_*x*_O_3_ (*x* = 0.00, 0.25, 0.50, 0.75, and 1.00), as shown in Fig. 1 (a–e). The refinement confirms the phase transition from rhombohedral to orthorhombic as the concentration of Fe-ions is increased in LaMnO_3_. Rhombohedral crystal structure with *R*3̄*C* space group was confirmed for 0.00 ≤ *x* ≤ 0.50 concentration of Fe-doping in LaMnO_3_, while the orthorhombic crystal structure with the Pbnm space group was obtained for 0.75 ≤ *x* ≤ 1.00. Different refined parameters along with their residual factors (*R*_p_, *R*_wp_, *R*_e_) are summarized in Table 1. The lattice parameters *a* = *b* = 5.5227 Å, *c* = 13.3646 Å and *a* = 5.5540 Å, *b* = 5.5637 Å, *c* = 7.8519 Å for LMO and LFO respectively, were found well-matched with the literature.^44,45^ Phase transition can be attributed to the ionic radii difference between Mn^4+^ (0.053 Å) and Fe^3+^ (0.064 Å) ions. Due to larger ionic radii of Fe^3+^ ions, as the Mn^4+^ ions are replaced by Fe^3+^ ions, it generates high pressure on the grain boundaries and results in structural transformation from rhombohedral to orthorhombic.

**Table 1:** Rietveld refined and calculated the structural parameters of LaMn_1−*x*_Fe_*x*_O_3_ ceramics

Simulated parameters			*x* = 0.00	*x* = 0.25	*x* = 0.50	*x* = 0.75	*x* = 1.00
			(Rhombohedral) (*R*3̄*c*)			(Orthorhombic) (*Pbnm*)	
Lattice parameters	*a* (Å)		5.5227	5.524194	5.52837	5.527039	5.5539
	*b* (Å)		5.5227	5.524194	5.52837	5.527039	5.5635
	*c* (Å)		13.3646	13.3869	13.4476	7.8535	7.8517
Volume of unit cell	*V* (Å^3^)		353.0115	353.7920	355.9479	241.033	242.6303
Atomic positions	La	*x*	0.0000	0.0000	0.0000	0.99211	0.99694
		*y*	0.0000	0.0000	0.0000	0.01920	0.02651
		*z*	0.2500	0.2500	0.2500	0.2500	0.2500
	Mn/Fe	*x*	0.0000	0.0000	0.0000	0.0000	0.0000
		*y*	0.0000	0.0000	0.0000	0.5000	0.5000
		*z*	0.0000	0.0000	0.0000	0.0000	0.0000
	O_1_	*x*	0.44736	0.45901	0.45901	0.75178	0.70561
		*y*	0.0000	0.0000	0.0000	0.70500	0.29975
		z	0.2500	0.2500	0.2500	0.0159	0.03019
	O_2_	*x*	—	—	—	0.09087	0.07964
		*y*	—	—	—	0.48723	0.47727
		*z*	—	—	—	0.2500	0.2500
*R*-factors (%)	*R*_p_		25.80	27.5	41.2	26.6	41.70
	*R*_exp_		16.67	12.6	19.7	12.7	18.34
	*R*_wp_		16.60	24.3	24.0	20.9	22.70
	*R*_f_		4.28	16.2	18.2	10.9	4.50
	*R*_Bragg_		1.930	6.28	2.43	4.88	2.664
GoF			1.00	1.87	0.45	1.97	1.53
Crystallite size (*D* (nm))			81.25	119.24	120.83	128.52	132.13
Lattice strain (*ε*)			5.58 × 10^−4^	2.94 × 10^−4^	3.24 × 10^−5^	−2.15 × 10^−4^	1.79 × 10^−4^
Dislocation density *δ* (nm^−1^)			1.52 × 10^−3^	7.03 × 10^−5^	6.84 × 10^−5^	6.05 × 10^−5^	5.73 × 10^−5^
X-ray density *ρ*x (g cm^−3^)			6.83	6.99	6.89	6.74	6.65
Specific surface area *S* (m^2^ g^−1^)			10.81	7.20	7.19	6.93	6.83

**Fig. 2:**
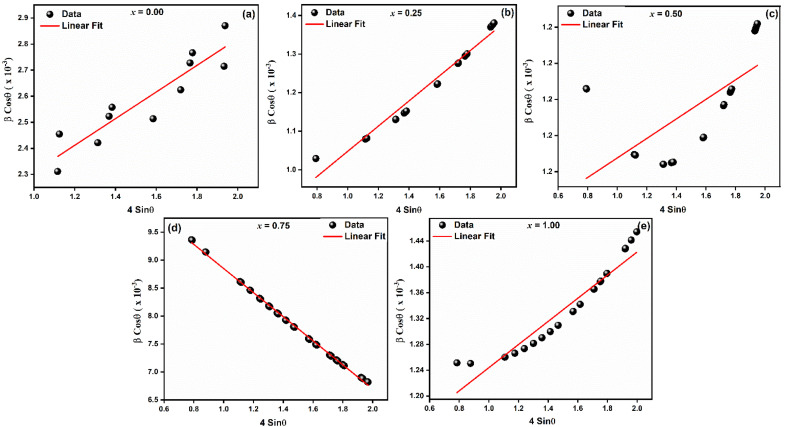
William–Hall plots of LaMn_1−*x*_Fe_*x*_O_3_ ceramics.

To investigate the kind of micro-strain seen in the crystal structures of LaMn_1−*x*_Fe_*x*_O_3_, the Williamsons–Hall (W–H) method was employed. To determine the crystallite size and micro-strain in the rhombohedral and orthorhombic crystal structures, the linear fitted line (as indicated in Fig. 2) was employed. For all samples except for compositions *x* = 0.75, which display a negative slope as illustrated in Fig. 2(d), the positive slope of the linear fit validates the tensile strain. The negative slope line confirmed the compressive strain. Because Fe-ions have large ionic radii, it is observed that when Fe-ions are doped in LaMnO_3_, the average crystallite size increases as the number of Fe-ions increases. The two intrinsic quantities that provide the dislocation network's measure are the lattice strain and the dislocation density. In Fig. 2(c) for *x* = 0.50 an exceptionally high value is observed. This may be due to the reason of equal concentration of Fe^3+^ and Mn^4+^-ions, which results into inhomogeneity at B-site. This inhomogeneity leads to lattice distortion due to the ionic radii of Fe^3+^ and Mn^4+^-ions. Therefore, maximum strain in the ceramic at *x* = 0.50 is observed. Fig. 2(e) a small deviations in the data point is seen, which may be due to the reason B-site is fully occupied by Fe^3+^-ions. Table 1 shows that a smaller lattice strain value corresponds to a lower concentration of lattice defects, whereas a reduced dislocation density corresponds to the production of a higher-quality sample. Eqn (1) and (2) were used to determine the synthesized NPs' X-ray densities (*ρ*_*x*_) and specific surface area (*S*).1
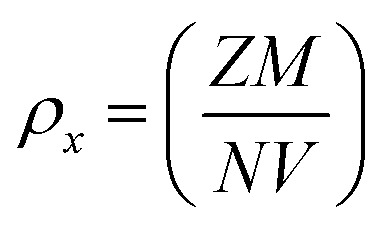

2 *S* = 6/(*Dρ*_*x*_)

where *M* is the samples' molecular weight, *N* is Avogadro's number, *V* is the unit cell's volume, and *D* is the average crystallite size, *Z* is the number of atoms in the rhombohedral/orthorhombic phase's unit cell. The obtained values of X-ray densities for these ceramics are listed in Table 1. The specific surface area (*S*) was found-to be decreasing with increasing crystallite size which can be-attributed to increasing surface-to-volume ratio.

**Fig. 3:**
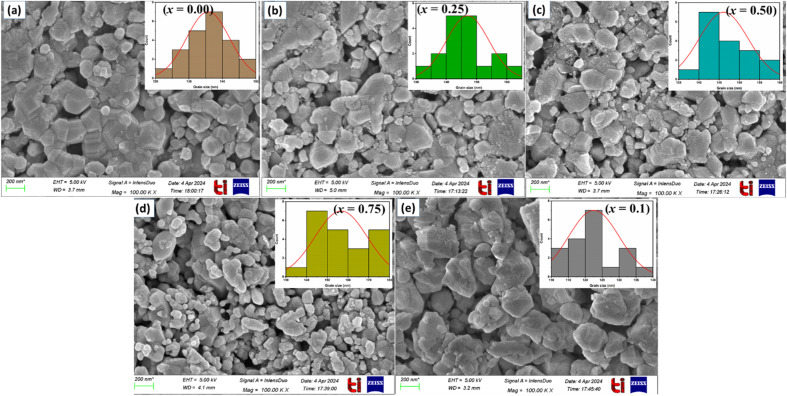
FESEM images of LaMn_1−*x*_Fe_*x*_O_3_ ceramics.

To well understand the microstructural features of LaMn_1−*x*_Fe_*x*_O_3_ (*x* = 0.00, 0.25, 0.50, 0.75, and 1.00), FESEM was performed as shown in Fig. 3. All the samples appear to contain quasi-spherical particles with shapes of similar uniformity. The microstructure consisted of sub-micron-sized particles with very fine morphology. All ceramics exhibit distinct, well-defined grains and boundaries.

Plotting histograms (inset) from SEM images using Image-J software allowed us to determine the average grain size, which ranged from 122 to 156 nm (see Table 2). The existence of secondary particles created by agglomeration accounts for the smaller average crystallite size found in SEM pictures compared to that found in X-ray diffraction.

**Table 2:** Elemental details and average grain size of LaMn_1−*x*_Fe_*x*_O_3_ ceramics

Composition	Elements	O	Mn	La	Fe	Grain size (nm)
*x* = 0.00	Wt (%)	18.68	23.14	58.18	—	135
	At (%)	58.15	20.98	20.98	—	
*x* = 0.25	Wt (%)	18.76	14.40	57.65	9.19	145
	At (%)	58.21	13.01	20.60	8.17	
*x* = 0.50	Wt (%)	17.61	4.28	57.63	20.48	146
	At (%)	56.14	3.98	21.17	18.71	
*x* = 0.75	Wt (%)	19.78	8.40	56.81	15.00	156
	At (%)	59.82	7.40	19.79	13.00	
*x* = 1.00	Wt (%)	18.92	—	55.50	25.58	122
	At (%)	57.97	—	19.59	22.45	

**Fig. 4:**
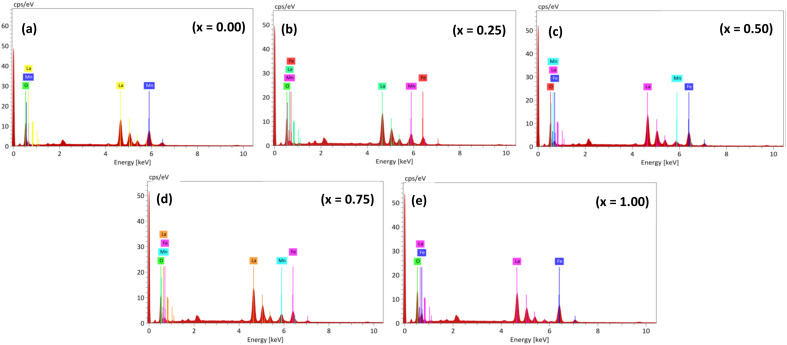
EDS spectra of LaMn_1−*x*_Fe_*x*_O_3_ ceramics.

EDS spectra confirm the homogeneous composition of the synthesized samples (as shown in Fig. 4), with elemental distribution mapped in LaMn_1−*x*_Fe_*x*_O_3_ (*x* = 0.00, 0.25, 0.50, 0.75, and 1.00). La, Mn, Fe, and O elements are detected at their expected energy levels, with atomic and weight percentages listed in Table 2, consistent with the nominal composition. No additional elements are detected, affirming the purity of the synthesized materials.

**Fig. 5:**
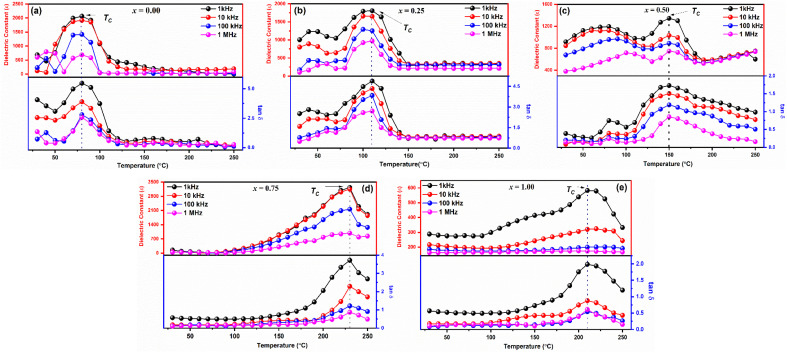
Temperature dependent dielectric constant (*ε*) and dielectric loss (tan *δ*) of LaMn_1−*x*_Fe_*x*_O_3_ ceramics.

The thermal variations of dielectric constants (*ε*) and dielectric loss (tan *δ*) in the temperature range 30–250 °C of the samples observed at frequencies 1 kHz, 10 kHz, 100 kHz, and 1 MHz, respectively are shown in Fig. 5(a–e). Typical dielectric dispersion behavior like ferroelectric materials has been observed in all the samples that is with increasing temperature the dielectric constants (*ε*) first increase and then attain a peak at Curie temperature (*T*_C_), and above *T*_C_, further increase in the temperature causes a rapid decrease in the dielectric constant (*ε*). It is noted that at low temperatures, *ε* is temperature independent and frequency independent for all samples. Then, for *x* = 0.00, 0.25, 0.50, 0.75, and 1.00, respectively, it increases progressively with increasing temperature to its maximum value (*ε*_max_), which corresponds to the change from a ferroelectric to a paraelectric phase, at approximately 60, 110, 150, 230, and 222 °C.

Moreover, dielectric constant decreases with increasing frequency as shown in Fig. 5(a–e). Change in *ε* values with frequency depends on the extrinsic as well as intrinsic contribution. For every sample, the contribution (extrinsic) from the grain boundary is greater than the bulk grain, as indicated by the huge values of *ε* in the low frequency. The Maxwell–Wagner model may provide an explanation for this, as the charges that build at the grain borders cause frequency-dependent behaviour that suggests the conducting grains and insulating grain boundaries that separate the polycrystalline perovskites. According to findings in the literature, the grain borders (extrinsic) contribute more at a low frequency than the grain contribution. The dielectric loss variation with temperature exhibits the same characteristics as the dielectric constant temperature variation, and it might be described using the same methodology as the dielectric constant discussion. It is discovered that the dielectric loss rises as the temperature climbs. Temperature-related increases in charge carrier mobility cause polarization to rise and significant dielectric loss. Charge accumulating at grain boundaries is the cause of the increased dielectric loss value that has been found at high temperatures.

**Fig. 6:**
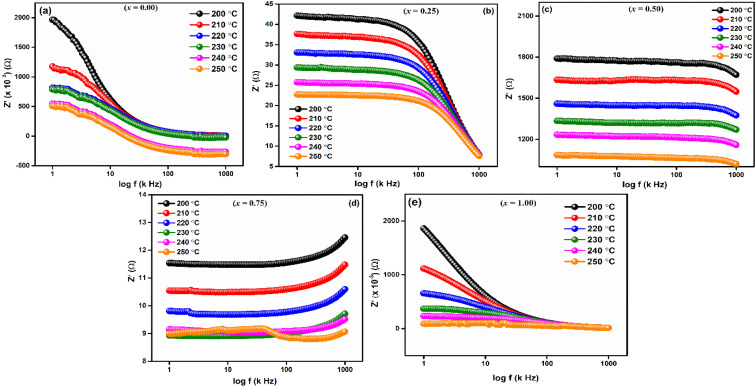
Frequency dependent real part of impedance (*Z*′) of LaMn_1−*x*_Fe_*x*_O_3_ ceramics.

Frequency-dependent of real part of impedance (*Z*′) is shown in Fig. 6 for LaMn_1−*x*_Fe_*x*_O_3_ (*x* = 0.00, 0.25, 0.50, 0.75, and 1.00) ceramics at high temperatures 200, 210, 220, 230, 240 and 250 °C, respectively. As shown in the figures, the magnitude of *Z*′ is higher at lower temperatures and decreases with increasing frequency for all compositions, confirming the NTCR behaviors of these ceramics. This decrease in *Z*′ corresponds to an increase in electrical conductivity. As frequency increases, *Z*′ values converge, leading to decreased barrier properties and the disappearance of polarization caused by space charge.^46^

**Fig. 7:**
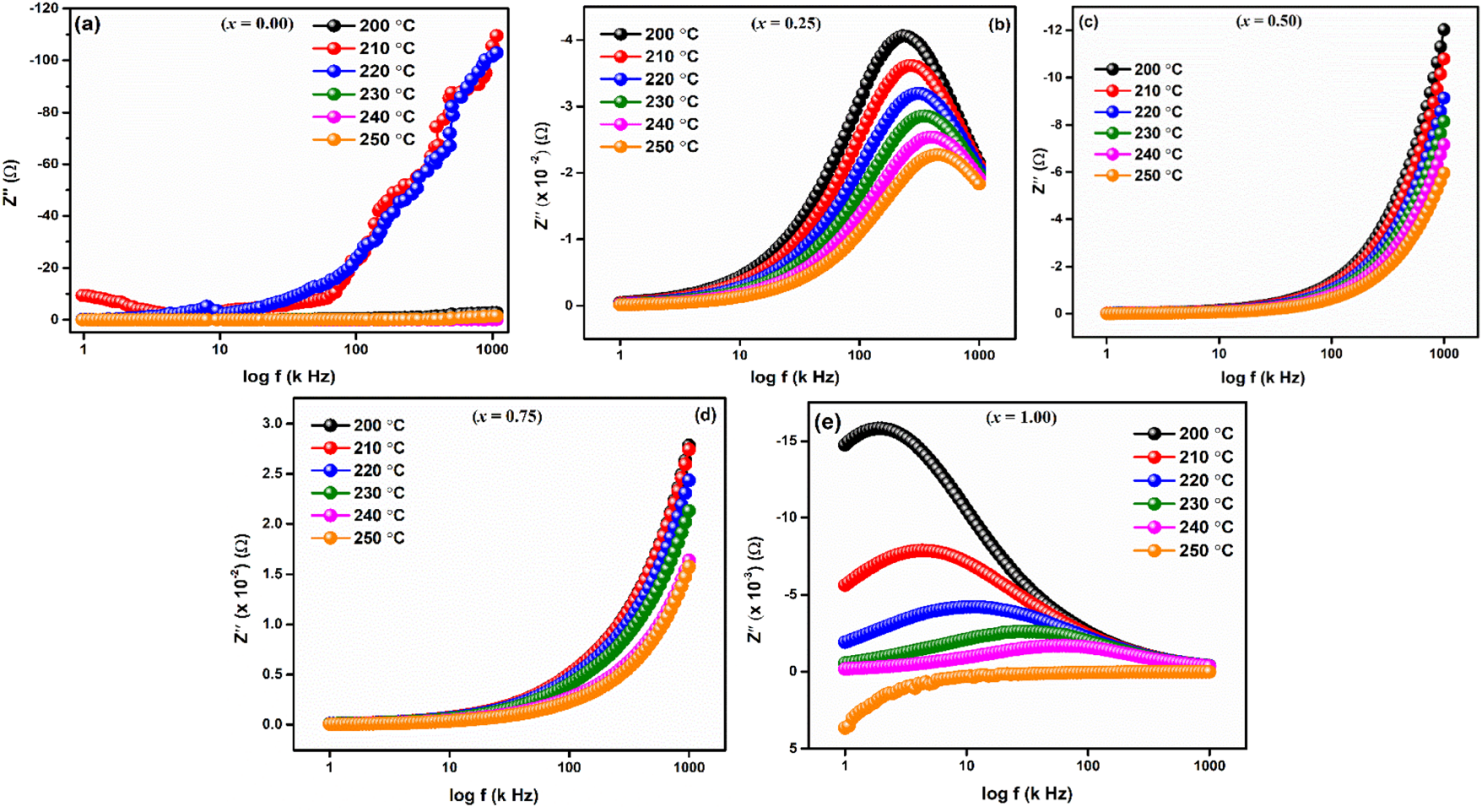
Frequency dependent imaginary part of impedance (*Z*′′) of LaMn_1−*x*_Fe_*x*_O_3_ ceramics.

Fig. 7(a–e) displays the imaginary portion of impedance (*Z*′′) frequency response curves for a wide range of temperatures (200–250 °C) for LaMn_1−*x*_Fe_*x*_O_3_ (*x* = 0.00, 0.25, 0.50, 0.75, and 1.00). Type and strength of electrical relaxation events in the system can be determined by looking for temperature-dependent peaks 
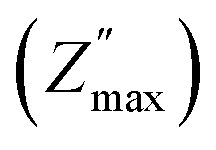
 at a specific frequency. The system's space charge is evident from this behaviour.^25,26^ As frequency and temperature rise, we observe that the value of (*Z*′′) first rises until peaking at 
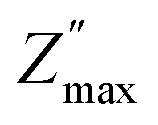
, after which it falls. The peaks shift towards higher frequencies as the temperature rises, which is a significant observation. It is significant to observe that as temperature rises, the peaks shift towards higher frequencies. The change in the peaks shows how the system's relaxation period is spreading, and the fact that this relaxation phenomenon evolved as the temperature rose supports the theory that temperature-dependent dielectric relaxation exists. The relaxation process, which causes electrical conduction in materials, may be caused by flaws created at higher temperatures. The fact that all compositions' height peaks or 
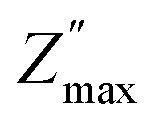
 values fall as temperature rises further supports the development of thermally activated charge carriers, which power the materials' conduction mechanism.^47,48^ The confirmation of a non-Debye-type relaxation process in the materials is provided by the asymmetric peak broadening, which reflects the relaxation time distribution. Furthermore, it was discovered that in the higher frequency area, all the curves combine at a particular frequency. This is thought to be caused by the space charge polarization decreasing at higher frequencies.^25–28^

**Fig. 8:**
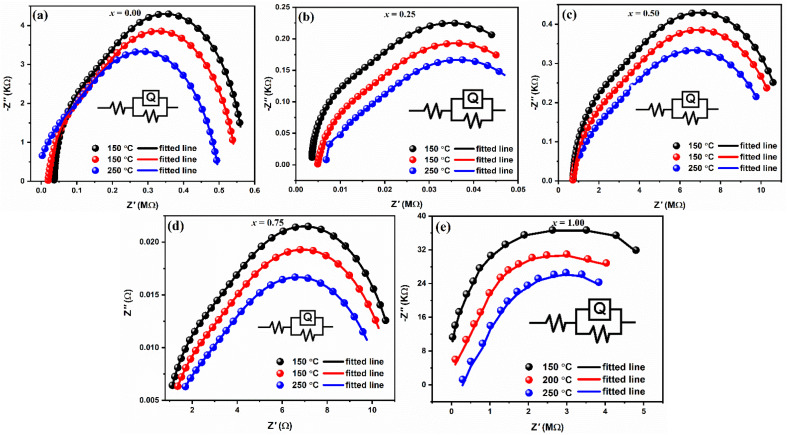
Cole–Cole plot for LaMn_1−*x*_Fe_*x*_O_3_ ceramics.

The Nyquist representation, which plots the imaginary part *Z*′′ *vs.* the real part *Z*′ in a Cartesian orthonormal reference frame, is one of the most used graphical representations in complex impedance spectroscopy. The literature^23^ states that the contribution of the grain boundary is represented by the semicircle in lower frequencies, while the semicircle in higher frequencies symbolizes the phenomena of intrinsic conduction, the response of the grains, and gives rise to the resistances of the grains. Fig. 8(a–e) shows our compound's Nyquist diagrams obtained at various temperatures. Each composition has a single, depressed semi-circular arc, its centre located below the real axis, signifying that it is semiconducting and has an NTCR. Every semicircle's diameter shrinks as temperature rises, suggesting a thermally triggered conduction process.^49^ The Nyquist plots are fitted with an equivalent circuit made up of two parallel resistances (*R*_g_ and *R*_gb_) and a constant phase element (*Q*) in order to verify the distinct contributions of grains and grain borders. Table 3 lists the fitted values resistance for grain and grain borders, whereas the black, red and blue lines show the fitted lines and dots shows the experimental data, respectively.

**Table 3:** Parameters calculated from impedance fitted data of LaMn_1−*x*_Fe_*x*_O_3_ ceramics

Compositions (*x*)	Temperature (°C)	Resistance		*n*	*Q*
		*R*g (Ωcm^2^)	*R*_gb_ (Ω cm^2^)		
0.00	150	2.0 × 10^−9^	2.1 × 10^4^	6.0 × 10^−1^	9.6 × 10^−10^
	200	1.5 × 10^−9^	2.3 × 10^3^	6.0 × 10^−1^	5.7 × 10^−10^
	250	1.0 × 10^−9^	2.3 × 10^3^	6.0 × 10^−1^	3.8 × 10^−10^
0.25	150	3.6 × 10^1^	3.3 × 10^3^	6.0 × 10^−1^	3.5 × 10^−9^
	200	4.7 × 10^2^	4.3 × 10^3^	6.0 × 10^−1^	1.1 × 10^−9^
	250	1.1 × 10^1^	2.9 × 10^2^	6.0 × 10^−1^	3.2 × 10^−9^
0.50	150	3.0 × 10^−7^	1.0 × 10^2^	6.0 × 10^−1^	7.8 × 10^−8^
	200	2.0 × 10^−7^	2.5 × 10^5^	6.0 × 10^−1^	2.8 × 10^−9^
	250	1.3 × 10^−7^	5.3 × 10^5^	6.0 × 10^−1^	5.9 × 10^−6^
0.75	150	2.5 × 10^−7^	1.2 × 10^9^	6.0 × 10^−1^	5.3 × 10^−7^
	200	3.0 × 10^−7^	1.6 × 10^4^	6.0 × 10^−1^	7.7 × 10^−7^
	250	1.6 × 10^−7^	2.3 × 10^6^	6.0 × 10^−1^	2.9 × 10^−7^
1.00	150	4.0 × 10^−7^	6.3 × 10^8^	6.0 × 10^−1^	8.6 × 10^−10^
	200	3.0 × 10^−7^	3.4 × 10^6^	6.0 × 10^−1^	2.8 × 10^−10^
	250	2.0 × 10^−7^	4.5 × 10^4^	6.0 × 10^−1^	1.9 × 10^−10^

**Fig. 9:**
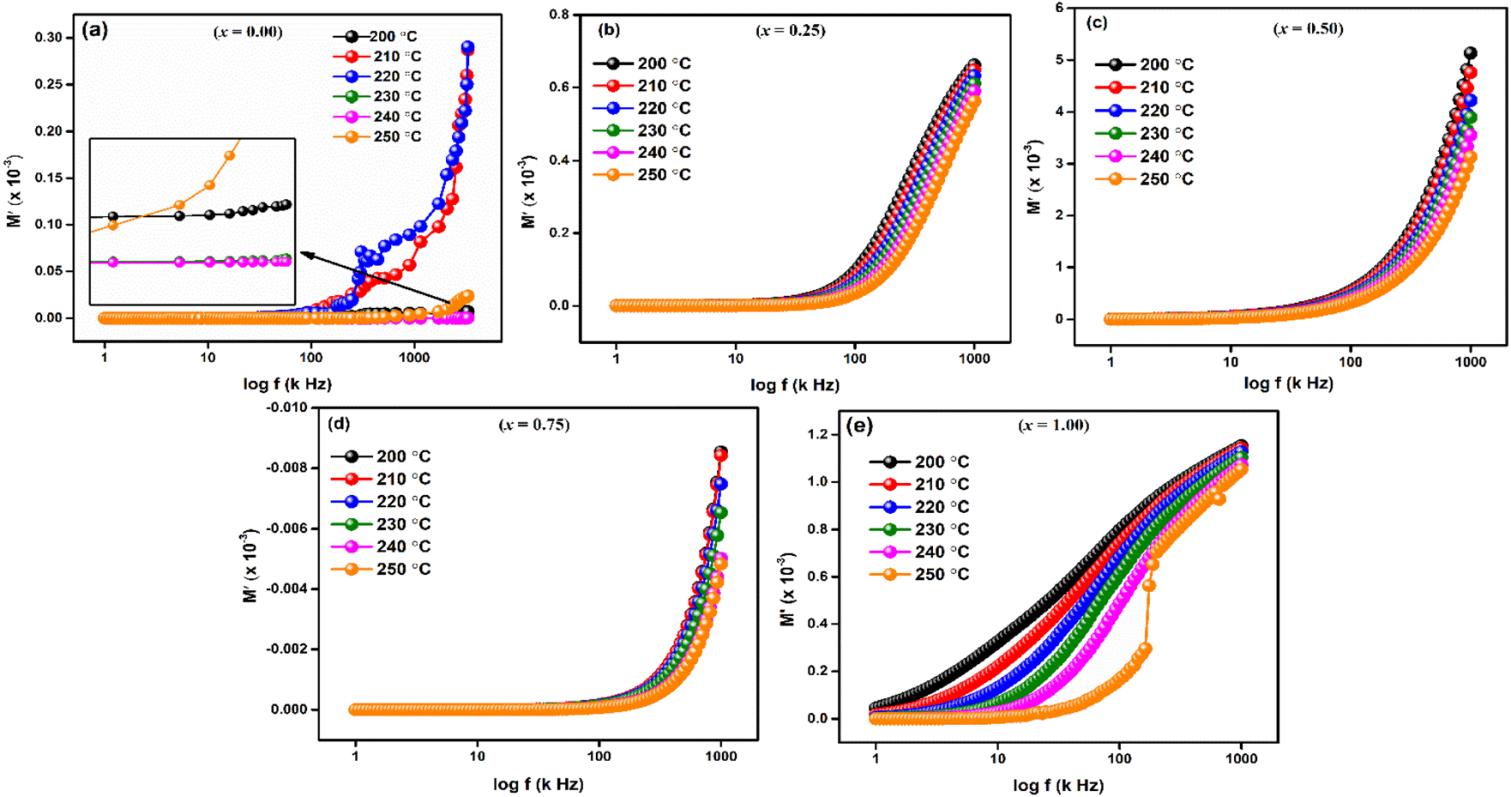
Frequency dependent real part of modulus (*M*′) of LaMn_1−*x*_Fe_*x*_O_3_ ceramics.

For LaMn_1−*x*_Fe_*x*_O_3_ (*x* = 0.00, 0.25, 0.50, 0.75, and 1.00) at temperature range 200–250 °C, Fig. 9(a–e) shows the frequency response of the real component of modulus (*M*′). The Figures show that for all compositions, the value of *M*′ is extremely low (near zero) in the low-frequency zone, and that it continuously increases as frequency increases, suggesting a tendency to saturate at a maximum temperature. The idea that the conduction process is caused by the charge carriers' short-range mobility is supported by this saturation characteristic.^50^ These explanations may relate to the idea that the mobility of charge carriers is brought about by an induced electric field when a restoring force isn't present. Because of the long-range mobility of charge carriers and the minimal impact of electrode polarization on the material, the low-frequency side's tiny value of *M*′ supports the conduction phenomenon.^51^

**Fig. 10:**
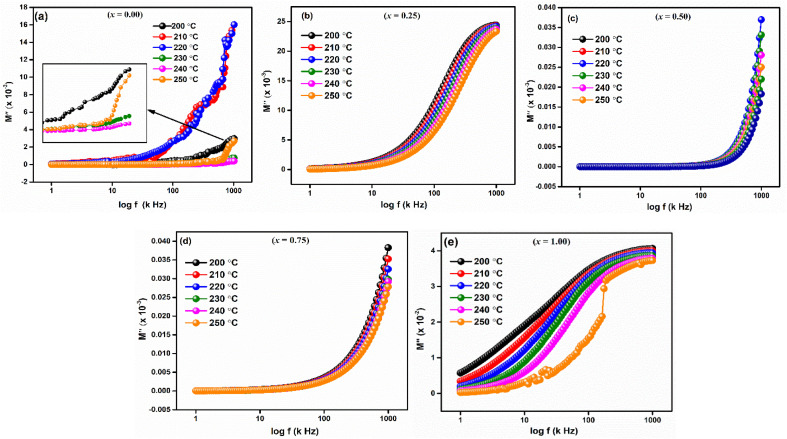
Frequency dependent real part of modulus (*M*′′) of LaMn_1−*x*_Fe_*x*_O_3_ ceramics.

The imaginary component of modulus (*M*′′) for LaMn_1−*x*_Fe_*x*_O_3_ (*x* = 0.00, 0.25, 0.50, 0.75, and 1.00) in a temperature range of 200–250 °C is displayed in Fig. 10(a–e). The relaxation peak shifts towards a higher frequency as the temperature rises. The observed asymmetry in peak broadening, which illustrates the spread of relaxation time with different time constants, supports the non-Debye type of relaxation in all the compositions.^52^

**Fig. 11:**
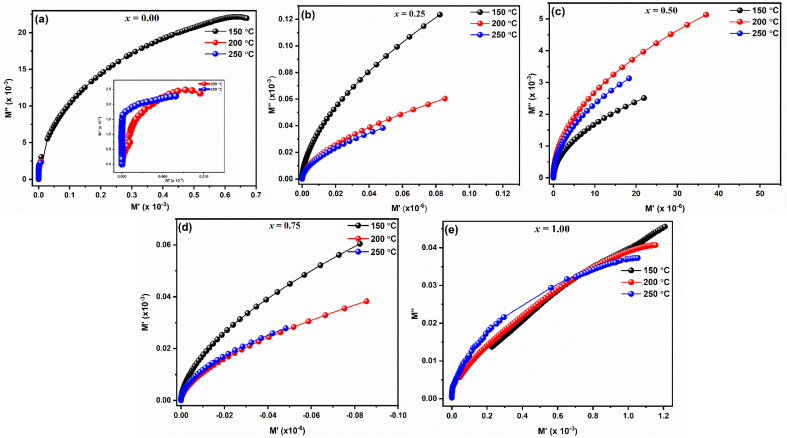
Modulus plots of LaMn_1−*x*_Fe_*x*_O_3_ ceramics.

Fig. 11(a–e) displays the complex electric modulus spectrum (*M*′′ *vs. M*′) for LaMn_1−*x*_Fe_*x*_O_3_ (*x* = 0.00, 0.25, 0.50, 0.75, and 1.00) at various temperatures. Electrical transport characteristics, including hopping rate and conductivity relaxation time, are interpreted by the electric modulus graph. By examining even, the tiniest changes in the materials' capacitance, it provides information on electrical processes. When it comes to differentiating the relaxation effects from grains (conducting regions) and grain borders (resistive plates) in materials, the *M*′′ *vs. M*′ plot performs better than the Nyquist plot of impedance (*Z*′′ *vs. Z*′). The phenomena with the smallest capacitance are identified by the modulus plot, while those with the most resistance is revealed by the impedance plot. Complex modulus analysis is suitable when two materials have similar resistances but differing capacitances.^53^ As seen in Fig. 11(a–e), the presence of a single semicircle for every composition whose centres are located below the real axis suggests that the grain effect predominates in the conduction mechanism over the grain boundaries effect.

**Fig. 12:**
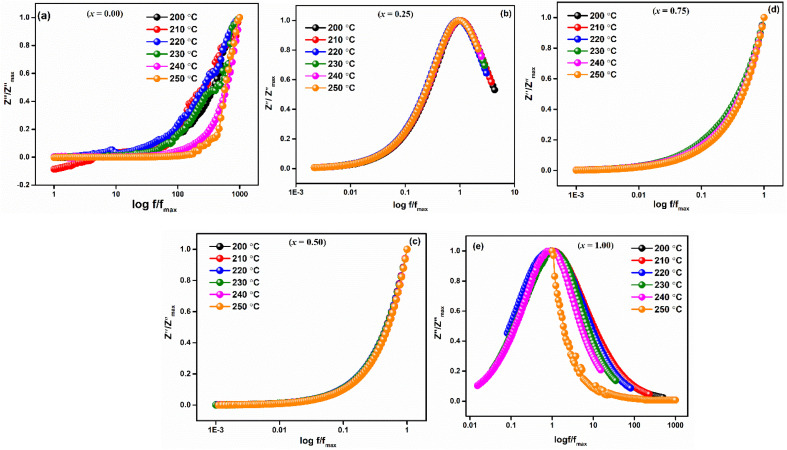
Master's plots of LaMn_1−*x*_Fe_*x*_O_3_ ceramics.

For every composition, a well-resolved semicircle at the higher frequency side is visible. This semicircle indicates the capacitive grain effect and shows that grains actively participate in the conduction mechanism. Furthermore, when temperature rises, a change in the grain semicircle's intercept on the *M*_x_ axis towards lower values of *M*′ is seen, suggesting an increase in capacitance that supports NTCR-type behavior. For LaMn_1−*x*_Fe_*x*_O_3_ ceramic compositions, the normalized plot of 
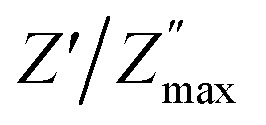
*vs.* log *f*/*f*_max_ at various temperatures nearly overlapped on a single master curve at various temperatures (Fig. 12(a–e)). The *Z*′′ peak frequencies exhibited a minor variation in full width at half maximum (FWHM) with an increase in temperature, but they still exhibited the same form and pattern at the peak position. The temperature-independent conduction transfer mechanism was confirmed by the master curve. Additionally, the non-symmetry of the curves was noted, suggesting that the conductivity relaxation did not behave in an exponential manner. 
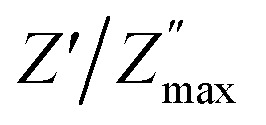
 FWHM as a function of log *f*/*f*_max_ was wider than a Debye peak's breadth, suggesting the existence of a relaxation process that is not Debye-type.

**Fig. 13:**
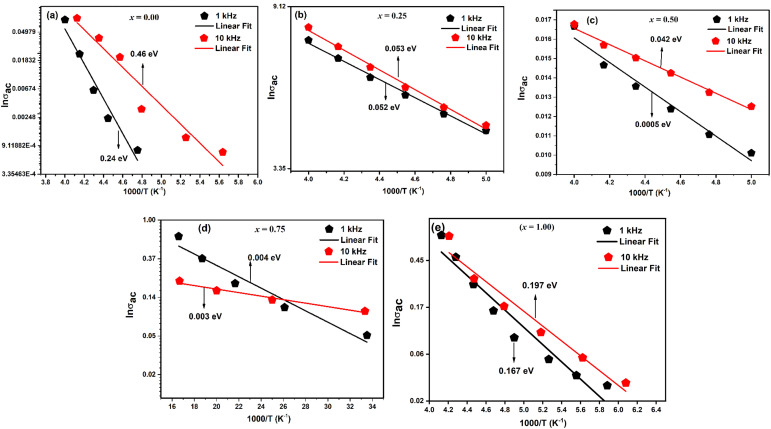
AC-conductivity as a function of 10^3^/*T* for LaMn_1−*x*_Fe_*x*_O_3_ ceramics (1 kHz & 10 kHz).

The fluctuation of ln(*σ*_ac_) with reciprocal temperature (10^3^/*T*) for compounds LaMn_1−*x*_Fe_*x*_O_3_ at certain frequencies (1 and 10 kHz) is displayed in Fig. 13(a–e). The NTCR behaviour of every sample is confirmed by the observation that the ac conductivity increases with temperature. Table 4 lists the activation energy of the relaxation mechanism for each sample. As the Fe content grew, the activation energy decreased, suggesting that the charge carrier concentration was hopping between the adjacent lattice sites more frequently. The obtained activation energies fell as the ceramic's Fe concentration increased, ranging from 0.24 to 0.167 eV. Because of the Fe substituent's larger lattice volume, the conduction-related specimens were released more readily and needed less energy to move.

**Table 4:** Comparison of the activation energy of LaMn_1−*x*_Fe_*x*_O_3_ ceramics at different frequencies with the selected literature

Ceramics	Composition (*x*)	Activation energy ((*E*_a_) eV)				References
LaMn_1−*x*_Fe_*x*_O_3_		1 kHz	10 kHz	100 kHz	1 MHz	This work
	0.00	0.24	0.46	0.39	0.22	
	0.25	0.052	0.053	0.052	0.166	
	0.50	0.0005	0.042	0.018	0.044	
	0.75	0.004	0.003	0.004	0.004	
	1.00	0.167	0.197	0.149	0.028	
La_0.8_Ca_0.15_Na_0.05_Mn_1−*x*_Fe_*x*_O_3_	0.00	0.173				54
	0.25	0.248				
	0.50	0.258				
	0.75	0.289				
La_0.7_Sr_0.3_Mn_1−*x*_Fe_*x*_O_3_	0.2	0.143				46
	0.3	0.108				
La_0.7_Sr_0.3_Mn_1−*x*_Fe_*x*_O_3_	0.08	0.135				33
	0.10	0.138				
	0.12	0.140				
La_0.7_Sr_0.3_Mn_1−*x*_Fe_*x*_O_3_	0.0	0.20				55
	0.10	0.19				
	0.15	0.17				
	0.20	0.16				
La_0.67_Sr_0.33_Mn_1−*x*_Fe_*x*_O_3_	0.05	0.073				56
	0.1	0.152				

**Fig. 14:**
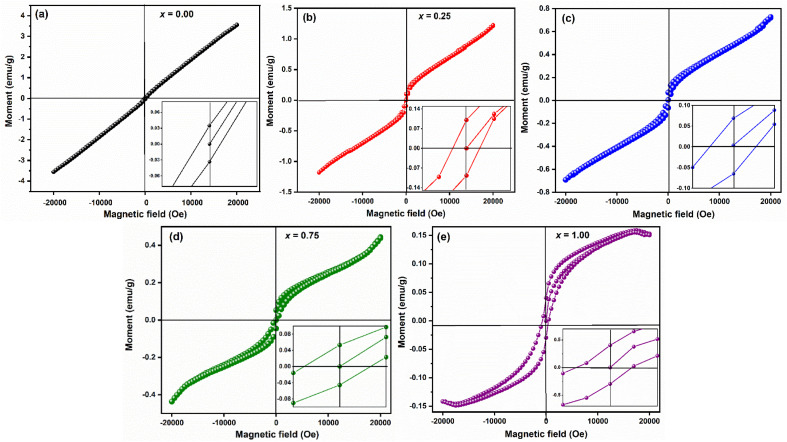
Room-temperature M − H loop of LaMn_1−*x*_Fe_*x*_O_3_ ceramics.

The room temperature hysteresis loop (HL) of LaMn_1−*x*_Fe_*x*_O_3_ is presented in Fig. 14. While La^3+^ is typically nonmagnetic due to paired electrons,^57^ the magnetic moments of Mn and Fe are responsible for magnetic ordering in this compound. Although LaMnO_3_ and LaFeO_3_ are known as antiferromagnetic materials, the HL of LaMn_1−*x*_Fe_*x*_O_3_ indicates weak ferromagnetism (FM) in all samples. The unsaturation of magnetization, even at high magnetic fields (20 kOe), suggests antiferromagnetic ordering. This weak ferromagnetism is attributed to antiferromagnetic order with canted spins, induced by the presence of a Dzyaloshinskii–Moriya (DM) interaction,^58^ which leads to a small magnetic moment in LMO and LFO NPs due to the spin canting of Mn and Fe ions.

Table 5 lists the estimated values of the various magnetic characteristics, including squareness ratio (SQR), coercive filed (*H*_C_), remanent magnetization (*M*_r_), and saturation magnetization (*M*_S_). The *M*_S_ value was found to be decreased from 3.55 emu g^−1^ to 0.14 emu g^−1^ with increasing Fe-concentration. The difference in magnetization values reflects the impact of small particle size resulting in a large surface-to-volume ratio for LaMnO_3_.

**Table 5:** Various magnetic properties of LaMn_1−*x*_Fe_*x*_O_3_ ceramics

Compositions	*M*_r_ (emu g^−1^)	*H*_C(if)_ (Oe)	*H*_C(df)_ (Oe)	*M*_s_ (emu g^−1^)	SQR = *M*_r_/*M*_s_	*μ*B
*x* = 0.00	0.035	84.23	159.39	3.55	0.01	0.15
*x* = 0.25	0.086	230.73	268.66	1.25	0.07	0.05
*x* = 0.50	0.069	293.38	295.21	0.73	0.09	0.03
*x* = 0.75	0.054	333.97	360.93	0.45	0.12	0.019
*x* = 1.00	0.033	491.50	696.08	0.14	0.24	0.006

On the other hand, coercive field (*H*_C_) was found to be increasing with increasing Fe-concentration, as bigger particles give a higher coercivity (*H*_c_ α *D*^6^). This is in good agreement with the law of the nano-magnetic particles. The coercivity value calculated at increasing and decreasing field (inset of Fig. 14, Table 5) indicated a shift in the HL around the origin, which confirms the presence of ferromagnetic/antiferromagnetic interfaces.^59^ The squareness ratio (*S*) of LaMn_1−*x*_Fe_*x*_O_3_ was found to be nearly equal to zero, while *H*_C_ ≠ 0, and *M*_r_ ≠ 0 which indicates that the prepared samples have particles with multiple domain sizes.

## Experimental

Polycrystalline LaMn_1−*x*_Fe_*x*_O_3_ (*x* = 0.00, 0.25, 0.50, 0.75, 1.00) were synthesized *via* high-temperature solid–state reaction method using high purity La_2_O_3_ (Sigma-Aldrich, 99.98%), MnO_2_ (Sigma Aldrich, 99%), and Fe_2_O_3_ (Merck, 99%) as the starting materials. The stoichiometric proportion of these oxides was weighted and mixed thoroughly (5–6 h) using mortar and pestle in the presence of hot distilled water as media. Following that, all of the sample reactant powders were calcined for five hours at 950 °C. Eqn (3) provides the following description of the suggested chemical reaction that would produce LaMn_1−*x*_Fe_*x*_O_3_.3
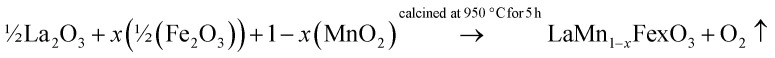


After the phase was confirmed by X-ray diffraction, a hydraulic press was used to form the powder into pallets with the appropriate dimensions. For improved densification, these green pellets were sintered for five hours at 1000 °C. Using CuK radiation (*λ* = 1.5406 Å) and a PANalytical X'Pert Pro Diffractometer at room temperature, the phase purity and crystal structure were once more examined. The surfaces of the sample were examined using Oxford Analytical instruments in combination with a Zeiss Sipra 55 field emission scanning electron microscope to determine micrographs, purity, composition, and chemical compositions. Using an LCR meter, dielectric characteristics were measured. The magnetic characteristics were measured at room temperature using a vibrating sample magnetometer (Microsense, Model: EZ9).

## Conclusions

A standard solid-state reaction technique was utilized to effectively synthesize LaMn_1−*x*_Fe_*x*_O_3_ (where *x* = 0.00, 0.25, 0.50, 0.75, and 1.00). Rietveld refinement of XRD patterns confirms a phase transition from pure rhombohedral to orthorhombic as Fe concentration increases in the LaMnO_3_ lattice. FESEM images reveal quasi-spherical grain morphology across all compositions. EDS spectra demonstrate elements present in stoichiometric ratios. Temperature-dependent dielectric spectra exhibit typical dispersion behavior, with high *ε* values at low frequencies attributed to grain boundaries, supported by the Maxwell–Wagner model. Complex impedance and modulus spectroscopy confirm thermally activated conduction mechanisms. Increasing AC conductivity with temperature confirms negative temperature coefficient resistance (NTCR) behavior. Activation energy, calculated from AC conductivity, decreases with higher Fe content due to increased lattice volume, easing specimen mobility with lower energy requirements. The magnetic hysteresis loop (HL) reveals weak ferromagnetism with antiferromagnetic (AFM) ordering, possibly due to Dzyaloshinskii–Moriya (DM) interactions inducing spin-lattice canting. Squareness ratios nearing zero suggest multiple domain sizes, correlating with increasing coercive field values. Based on the above-received properties these ceramics are suitable for the spintronic applications.

## Future research scope

This study focused on the experimental tailoring of structural, morphological, electrical, and magnetic properties of LaMn_1−*x*_Fe_*x*_O_3_ ceramics, future research could greatly benefit from the integration of Density Functional Theory (DFT) calculations. DFT could provide detailed insights into the electronic structure, predict various material properties, and assist in understanding the behavior of dopants at the atomic level. For instance, DFT calculations could help elucidate the electronic band structure and density of states, providing a deeper understanding of the electrical conductivity mechanisms in these ceramics. Additionally, it could offer predictions on the magnetic interactions within the material, guiding further experimental investigations.

A recent study by Wang *et al.* has demonstrated the utility of DFT in understanding competitive adsorption behaviors in environmental contexts, specifically on the facets of Goethite.^60^ This approach could be similarly beneficial in studying LaMn_1−*x*_Fe_*x*_O_3_ ceramics, where DFT could help in understanding the coordination structure affinity of different dopants and their influence on the material's overall properties.

The tailored properties of LaMn_1−*x*_Fe_*x*_O_3_ ceramics open up a wide range of potential applications. Due to their tunable electrical and magnetic properties, these materials could be used in electronic devices such as sensors, actuators, and memory devices. Their unique magnetic properties also make them suitable for applications in magnetic storage media and spintronics. Additionally, the structural and morphological versatility of these ceramics could be exploited in catalysis and other industrial applications where specific surface characteristics are crucial. In conclusion, integrating DFT calculations with experimental research could significantly advance the understanding and development of LaMn_1−*x*_Fe_*x*_O_3_ ceramics, enabling the optimization of their properties for various high-performance applications.

## Data availability

Data will be made available on reasonable request by corresponding author.

## Author contributions

P. T.: methodology, writing-original draft, software, visualization. K. I. N.: writing-review & editing. D. K.: writing-review & editing. P. K.: writing-review & editing. P. S.: resources. V. T.: writing-review & editing. A. S. A.: writing-review & editing. A. A.: writing-review & editing. M. E.: writing-review & editing. M. L.: conceptualization, software, visualization, data curation, supervision, writing-review & editing.

## Conflicts of interest

The authors declare that they have no known competing financial interests or personal relationships that could have appeared to influence the work reported in this paper.
